# Understanding the Sub-Cellular Dynamics of Silicon Transportation and Synthesis in Diatoms Using Population-Level Data and Computational Optimization

**DOI:** 10.1371/journal.pcbi.1003687

**Published:** 2014-06-19

**Authors:** Narjes Javaheri, Roland Dries, Jaap Kaandorp

**Affiliations:** 1 Section Computational Science, University of Amsterdam, Amsterdam, The Netherlands; 2 FOM Institute AMOLF, Amsterdam, The Netherlands; University of Bristol, United Kingdom

## Abstract

Controlled synthesis of silicon is a major challenge in nanotechnology and material science. Diatoms, the unicellular algae, are an inspiring example of silica biosynthesis, producing complex and delicate nano-structures. This happens in several cell compartments, including cytoplasm and silica deposition vesicle (SDV). Considering the low concentration of silicic acid in oceans, cells have developed silicon transporter proteins (SIT). Moreover, cells change the level of active SITs during one cell cycle, likely as a response to the level of external nutrients and internal deposition rates. Despite this topic being of fundamental interest, the intracellular dynamics of nutrients and cell regulation strategies remain poorly understood. One reason is the difficulties in measurements and manipulation of these mechanisms at such small scales, and even when possible, data often contain large errors. Therefore, using computational techniques seems inevitable. We have constructed a mathematical model for silicon dynamics in the diatom *Thalassiosira pseudonana* in four compartments: external environment, cytoplasm, SDV and deposited silica. The model builds on mass conservation and Michaelis-Menten kinetics as mass transport equations. In order to find the free parameters of the model from sparse, noisy experimental data, an optimization technique (global and local search), together with enzyme related penalty terms, has been applied. We have connected population-level data to individual-cell-level quantities including the effect of early division of non-synchronized cells. Our model is robust, proven by sensitivity and perturbation analysis, and predicts dynamics of intracellular nutrients and enzymes in different compartments. The model produces different uptake regimes, previously recognized as surge, externally-controlled and internally-controlled uptakes. Finally, we imposed a flux of SITs to the model and compared it with previous classical kinetics. The model introduced can be generalized in order to analyze different biomineralizing organisms and to test different chemical pathways only by switching the system of mass transport equations.

## Author Summary

Understanding complex biological systems, especially at intracellular scales, has always been a big challenge, owing to the difficulties in measuring and manipulating such small quantities. Computational modeling brings promising possibilities to this area. The model organism we have studied here is the diatom, a single cellular silicifying organism. Diatoms live in most water habitats and they use the very low concentrations of silicon in the oceans to develop beautifully complex silica structures. The cell control strategies acting on this process have been a long-standing open question. In this work, we have modeled the silicon uptake, transport and synthesis in diatoms in different cell compartments. To find the best set of free parameters of the model we solved the inverse problem using parameter identifiability, global optimization, sensitivity and perturbation techniques. The resulting model is a framework for manipulating and testing different properties of cells; for example, we have tested the cell control on silicon uptake by changing the expression level of the transporter proteins. Such modeling, described in this work, is both a necessary and important tool for understanding the cell strategies over control of material transport and synthesis.

## Introduction

Every cell has at least one membrane to separate it from the outside environment and to make it *a living unit*. The existence of a membrane and the division of the cell space (compartments) causes a discontinuity in the distribution of materials and energy between the inside and the outside of the cell. This introduces an effective control on cell preferences for material synthesis and energy production/consumption. Moreover, the membrane borders have an effect on the geometry of structures inside them. One of common means for this control is through the production of specific proteins that act as transporters. The mass transport through different cell compartments is a universal phenomenon and, since it has a significant effect on all subsequent intercellular processes, it attracts great interest for compartmental modeling studies [1]–[3].

One interesting example of compartmental strategies by cells for control of material synthesis can be found in biomineralizing organisms that make specific pattered minerals with the assistance of biomolecules [4]–[6]. Examples of this are iron oxides and sulfides in bacteria, silicates in diatoms, carbonates in algae, and calcium phosphates and carbonates in vertebrates [7], [8]. They uptake, synthesize and consume minerals in favor of producing hard skeletons in combination with an organic matrix (hybrid materials).

### Diatoms

Diatoms are the eukaryotic unicellular organisms, living in marine and fresh water environments. Diatoms are producers in the food chain. They generate around 40% of all organic carbon in the sea [9]. They contribute to the global carbon cycle, by photosynthetic carbon fixation as much as all the terrestrial rain forests do [10], [11] and therefore, it's likely that they have influenced the global climate over millions of years, by the inward flux of carbon dioxide to the oceans [12]. Moreover, they are major players in the silicon cycle of the oceans [13].

**Figure 1:**
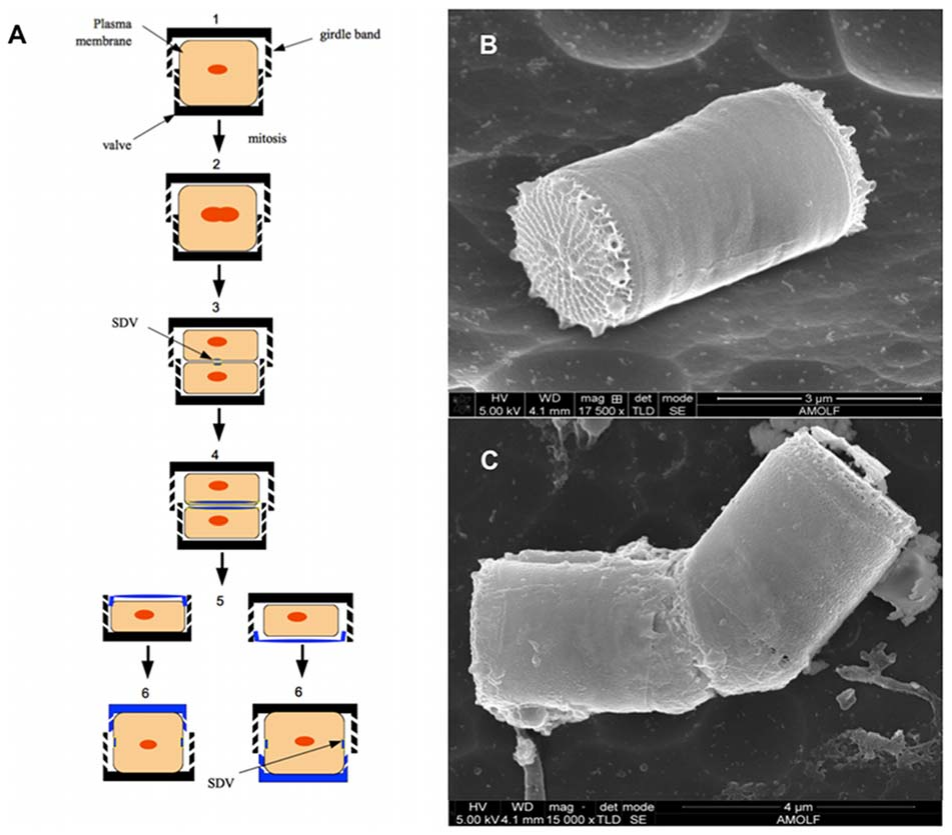
Cell cycle and cell division of diatoms. (A) Schematic picture of different stages of the asexual cell cycle of a typical diatom: Silica wall is composed of valves and girdle bands. 1: a full-size diatom cell, 2: Cell during the DNA replication (S-phase), 3; immediately after this silica deposition in SDV starts, 4&5: the valve formation continues until it is ready for the new sibling cells and then the cell divides (G2+M-phase), 6: new cells grow accompanied with silica girdle bands formation (G1) until it achieves the full size. (B)&(C) SEM images of diatom *Thalassiosira pseudonana*. The image of silica frustules of one diatom including valves and girdle bands (B) and a diatom during cell division (C).

The distinctive feature of diatoms is that they synthesize a wall around themselves, called frustule, made of amorphous silica 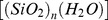, which is delicately patterned down to the scale of nanometers. The frustule structure varies between different species of diatoms and also between different growth conditions. [14]–[16]. The precision in controlled mineralization is one of the reasons that they have been attracting increasing interest for a long time, from a material science perspective [14], [17]–[21]. The oldest diatom fossils have been dated back to 185 Mya [22]. It is suggested that the abundance and ecological success of diatoms can be a result of their silica walls - living in a “glass house” [23], [24]. Diatom frustules are typically composed of two parts: the valve, which forms the larger outer surface and girdle bands, which are rings of silica being produced during cell growth (Figure 1A).

The necessity of silica formation for diatom cells imposes special steps in their cell cycle. Figure 1A depicts the asexual cell cycle of a typical diatom. Before a cell divides, new silica valves for the next generation are formed inside a specialized vesicle, the Silica Deposition Vesicle (SDV) [25], [26]. After this event, daughter cells separate and start growing, which includes the growth in the cell volume and also the silica girdle bands until the cell reaches its largest size.

If the amount of silicon in the environment is depleted to almost zero; most cells will be arrested in the G1-phase or G2-M phase, with the G1 arrest point being generally predominant [27]–[29]. This property has been very useful in diatom studies because under silicon starvation condition, cells become mostly synchronized in their cell cycle and therefore, an individual cell can be studied easier with the data from population-level quantities. However, even in the best case usually up to 80% of the cells are synchronized and, therefore, there will be an error in downscaling to a single cell dynamics from macroscopic quantities.

Because of the specific cell division form, including one valve growing inside another, in many species of diatoms, one of the daughter cells becomes smaller after each generation. To overcome this problem, after many generations, cells that are smaller than a critical threshold will be regenerated to the original size via sexual reproduction [30]. For some species of diatoms, however, sexual reproduction has never been observed. [31],[32]. A study on the frustule formation based on fluorescence imaging of the species *Thalassiosira pseudonana*, suggests that sometimes girdle bands close to the cell center expand and are thus able to accommodate a new valve, which is not smaller than parent's valves [29].

**Figure 2:**
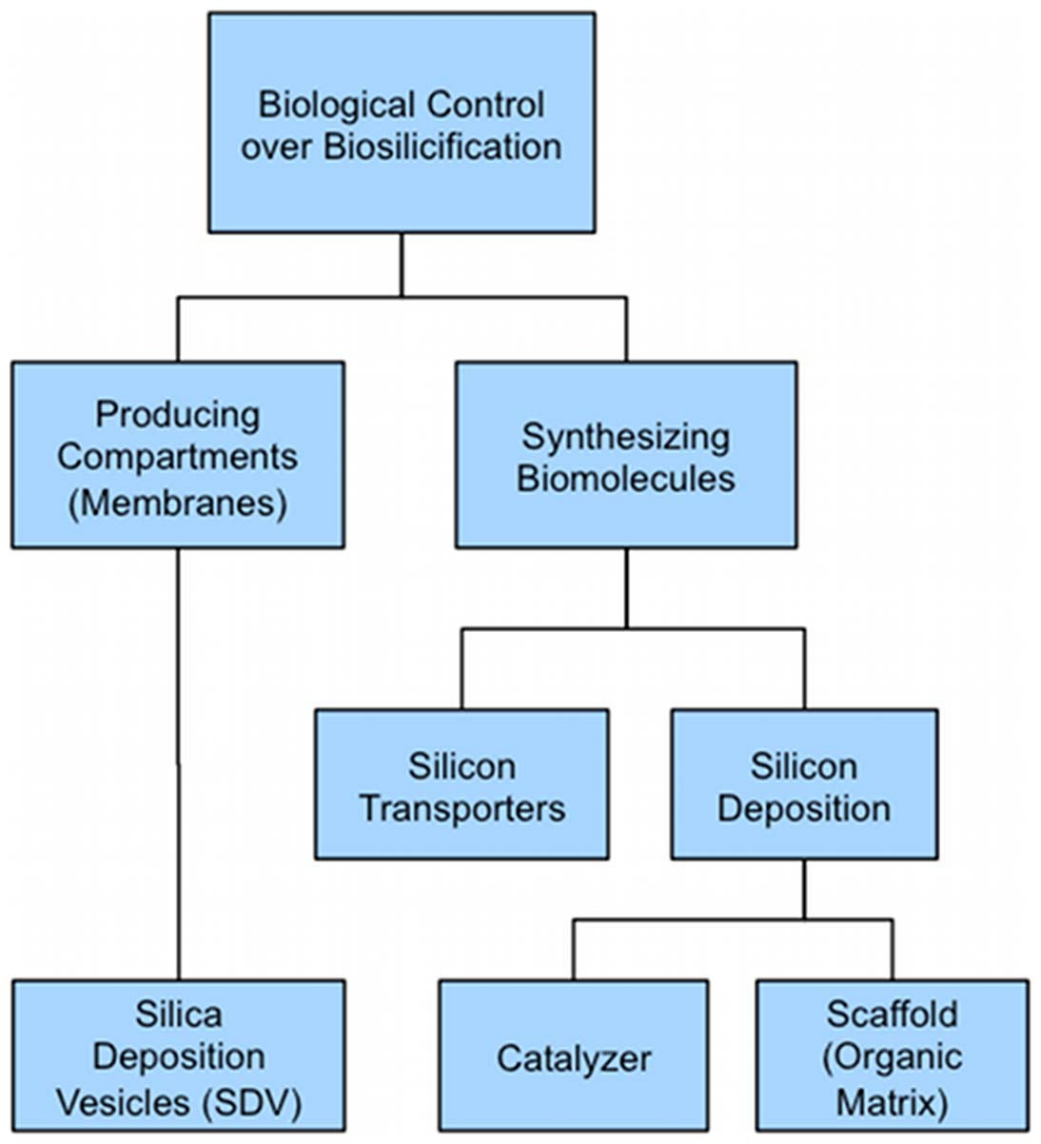
Biological control over biosilicification. Cell controls mineralization through two types of mechanisms: By formation of membranes in order to make specialized compartments like SDV and by producing biomolecules such as silicon transporter proteins and biomolecules involved in silica deposition. The latter includes molecules with the role of scaffold or catalyzer.

The silica wall architecture is a species-specific characteristic of diatoms, which is an indication that the synthesis of silica is highly genetically controlled, in addition to being chemically and physically controlled. Since the entire genome of some species of diatoms has been sequenced (*Thalassiosira pseudonana*: [33] and *Phaeodactylum tricornutum*: [34]), there have recently been greater insights into this genetic control [35],[36]. Figure 2 is a diagram showing cell control mechanisms. It is believed that such control takes place mainly through two processes. Firstly, via synthesizing and providing special membranes like SDV and secondly, via producing functional biomolecules. The first type of silicification-related biomolecules regulate uptake and transport of silicon and the second type are involved in deposition of silica including proteins and polyamines, which play the role of catalyst or a structure forming scaffold [4],[5],[37]–[39].

Our model organism is *Thalassiosira pseudonana*. Figure 1B and 1C shows a scanning electron microscope (SEM) image of this species. In Figure 1B the silica structure of valves, which is specific for this species, and girdle bands, which have a finer and more regular structure, are presented. Figure 1C shows the image of a diatom captured during the last step of cell division. It is clear in this image that the daughter cells already have completed valves before division takes place.

### Silicon uptake

Diatoms uptake silicon mostly in the form of neutral orthosilicic acid Si(OH)_4_ [40],[41]. The low concentration of silicic acid in present oceans might be the reason for the development of active transportation. Cells synthesize special silicic acid transporter proteins (SITs) to act on the membrane for making an inward silicon flux in the cell. The role of SITs has been discussed in several studies [e.g. 42, 43].

In *T. pseudonana* three distinct SIT genes have been analyzed for their regulatory mechanisms. It has been shown that SIT protein levels change during a synchronized cell cycle (up to 50% changes around an average value) and that the peaks of their profile occur during silica deposition periods of the cell cycle. Moreover, the peaks in mRNA levels happen in S-phase, where the period prior to valve formation shows the highest uptake rate [44]. This causes non-classical enzyme kinetics, which is when the kinetic coefficients are time-dependent in contrast to classical enzyme kinetics when the coefficients are assumed to be constant. In this case, the maximum uptake rate is not constant, but it is a dynamic quantity due to the flux of enzyme production and dynamic enzyme activities [44]. This effect changes the chemical pathways [45]. More interestingly, the SIT3 mRNA level is much lower than SIT1 and SIT2 and also SIT3 is not up-regulated in response to silicon starvation or cell cycle as much [33],[44]. This suggests that SIT3 might act as a sensor for external silicon concentration [46]. The sensor role of some proteins has been observed in other cells like yeast (e.g. [47]). In that case when the nutrient concentration is lower than a threshold a different type of transporter with a high affinity is produced. This behavior is associated with a dual-transport system, which has been shown in the case of yeast that it prolongs the preparation for starvation and it facilitate the subsequent recovery of cells [47].

### Silicon storage and pre-synthesis in diatoms silicon pool

After cell uptakes silicon it stores it partly in a soluble silicon pool and then transports it through cytoplasm to reach the SDV, the location of the new synthesizing walls. Currently, the intracellular location of the pool and the mechanisms by which it transports to SDV is not completely understood. Interestingly, if we consider all the intracellular silicon pool in the form of monosilicic acid, considering the small volume of the cell, it should have a concentration higher than the solubility of monosilicic acid, which is around 2mM at pHs below 9 [48]. It has thus been an open question as to how the cell can maintain this concentration without deposition. There are different scenarios for explaining the storage and transport of silicon in the pool. One of these scenarios assumes that the silicic acid binds to some type of organic molecule and thus makes a soluble silicon pool [49] and therefore it turns the silicic acid into another chemical form. This explanation is in agreement with the uptake behavior of diatoms after different starvation conditions [50]. Another scenario assumes the existence of special silicon transport vesicles (STVs), which transport silicic acid from the cell membrane and release their content into SDV by fusing to its membrane [51], however, the existence of silicon inside such vesicles, to the best of our knowledge, has not been proved. A third scenario shows that oligomerization indeed starts inside the cell as soon as there is some monosilicic acid available in cytoplasm, generating precursors for later deposition inside SDVs. This explanation is based on NMR data from silicon pools (see the following).

Recently, the NMR chemical shift technique has been a powerful way for understanding the forms of silicic acid based on their connections to other molecules. With this data it has been shown that the majority of silicon in the entire cell is in a polymerized form [52]. However, this data does not necessarily exclude the possibility that the first explanation could have a role (because this method cannot distinguish between free monosilicic acid and its attachment to an organic molecule), but it shows clearly that intracellular silicon is mostly in the form of oligomers. Finally, after the nutrient (oligomers of silicic acid and pre-synthesized silica) is provided for SDV, silica precipitation and pattern formation occurs.

In this study, we model silicon uptake, transportation and synthesis in diatoms. We use the nonlinear Michaelis-Menten kinetics for protein activities as mass transport equations. The model is composed of four compartments: external environment, cytoplasm, SDV and deposited silica. In order to find the unknown parameters of our compartmental model, scatter search, (a global optimization technique), has been applied for fitting the model to experimental data on silicon consumption and cell population growth. To add constraints to the model, a penalty term is added to the objective function. Additionally, the optimized solutions have been tested with sensitivity and perturbation analysis. The resulting robust optimized solutions predict silicon dynamics and intracellular biosilicification rates which are in agreement with experimental measurements. With the insight from SITs expression level data, we then impose a flux of proteins during the cell cycle and investigate its effect on silicon uptake rates.

## Materials and Methods

### Compartmental modeling

In order to model the distribution and transportation of materials in cells, two computational methods have been commonly used. In the first method, spatial and temporal quantities are both important and a system of PDEs should be solved with some degree of accuracy. However, in many cases, cell processes are not diffusion-limited, meaning that the rate of another event that plays the role of a sink or a source, is slower than diffusion rate and thus diffusion is fast enough to keep the distribution of materials homogenous in the time-scale of interest.

To investigate the effect of diffusion on transport of silicon two types of diffusion rates have been calculated. For the detailed calculation see Text S1 from supplementary materials. The first type is diffusion through membrane. Even though the membrane is semi-permeable for monosilicic acid, which is a small, uncharged molecule, the rate of diffusion is much smaller than the observed uptake rate [53]–[56]. Therefore, it seems that active transporters are the most important method for the transport. The second type of diffusion is responsible for distribution of materials close to the membrane in each of its sides. In text S1 we show that diffusion of silicic acid in water is fast enough compared to the rate of uptake, that it will not be a controlling factor (See Figure S2 in Text S1). The diffusion-mediated Michaelis-Menten equation also shows that the effect of diffusion on the total rate is negligible [45]. This allows us to use compartmental modeling as a good approximation.

If cells have several compartments, the chemical and physiological characteristics of inside and outside compartments can differ significantly. This is an efficient mechanism for regulating special events and as expected it requires energy for synthesizing new biomaterials for membranes. In such a process that is not diffusion-limited the second modeling approach, compartmental modeling, is suitable. Compartmental modeling includes a system of ODEs that describes the dynamics of materials inside and its transportation between compartments.

There are several reasons that the uptake of silicon, in the form of mono silicic acid, is mostly controlled by enzymatic reactions. For instance, cells use the so-called SIT (silicon transporters) genes to produce proteins with active uptake sites and subsequently lower the energy barrier for silicon uptake through membranes. Although in most studies of SITs, the focus is on the SITs on cell membranes, but the expression level and other relevant data usually is derived from the whole cell proteins analysis. Since in diatom cells, there is a specialized compartment for silica synthesis and deposition which is surrounded by a membrane, there is no reason why this membrane shouldn't be equipped with SITs; either in the same form or a similar form. Moreover, the permeability of intracellular membranes to silicic acid is low enough that transporter proteins still are likely the major way of transportation [53].

**Figure 3:**
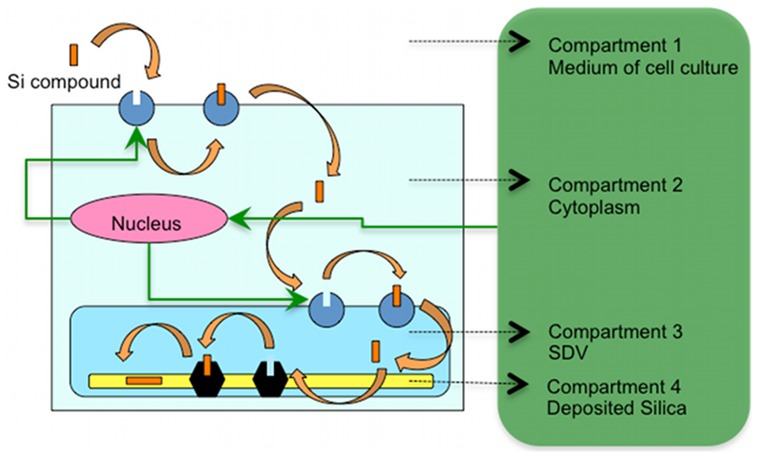
Four compartments considered in the computational modeling and kinetics of their communication. Compartment 1 is the environment from which diatom uptakes silicic acid by means of silicon transporter proteins (SIT) located on the cell membrane. Then silicon most likely stored in compartment 2, the cytoplasm, and transported to SDV, the compartment 3, again by means of SITs. In SDV the soluble silicon finally deposits and forms the silica frustule, which is compartment 4. Green arrows show the cell regulation over amount of SITs. Once the cell nucleus receives the related signals based on information from inside and outside the cell, with some mechanisms which are mostly unknown, it changes the level of SITs to control the inward flux of silicon to compartments. (This figure is a schematic graph. In reality the location of SITs is not completely clear.)

Our compartmental model includes four compartments (see Figure 3) and we aim to model the temporal changes in amount of silicon (mol), regardless of the form of the silicon compound, inside those four parts. Once silicic acid is inside the cell, it will likely undergo polymerization [52]. The silica polymerization chain of reactions contains many reaction terms, giving rise to a high degree of complexity. Moreover, there is not much known about the oligomer form of silicic acid in diatoms. Therefore, a useful and applicable approach is to consider the total amount of silicon in each compartment and calculate the temporal changes of this quantity.

In order to include the deposition of silica in the SDV, we consider deposited amorphous silica as one compartment. Biological molecules, which consist mainly of proteins and long chain polyamines (LCPAs), have a major role in silica polymerization and pattern formation [35]–[39]. The process by which they guide biomineralization is studied to some extent but still not completely understood. Moreover, there are a variety of complex events happening in SDV, including self-aggregation of organic molecules and organic matrix formation, polymerization of silicic acid and formation of oligomers both from monosilicic acid and also from precursors of oligomers, nucleation and phase separation and formation of silica under a controlled condition of concentrations and pH values. Also those events have different time and spatial scales [4]–[6]. It is apparent that the entire process is too complex to study it in a single model. In our model, we present the equilibrium condition of the SDV membrane to be described by a catalytic process. Moreover, we introduce the phase separation event, the transport between the soluble and deposited silica in SDV, as the transport between compartments 3 and 4.

Figure 3 also shows the cell control over level of SITs, which is regulated in response to changes in external concentrations of silicic acid and internal consumption of silicon, as discussed earlier in the introduction. We introduce this control in one of our experiments by adding a flux term to the rate equation of the SITs.

In this model the focus is on uptake and transportation of silicic acid through the cell membrane and cytoplasm. This step is vital in providing the material for the silica depositing compartment and, consequently, vital for the cell division.

### Kinetics of mass-transportation – Forward problem

#### Relation between individual-cell-level and population-level quantities: A multiscale problem

To model a biological system containing a population of cells and a common medium we need equations describing the dynamics of the medium and the dynamics of individual cells. This could be written as equations (1) and (2).
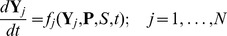
(1)




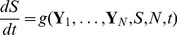
(2)



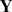
 is a vector containing all dependent variables of intracellular dynamics like concentrations, 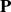 is a vector containing all the independent parameters of the intracellular model, 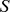 is the external nutrient concentration, and 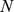 is the total number of cells. Equation (1) describes the intracellular dynamics of cell j, which is a function of internal and external quantities. On the other hand, equation (2) describes the nutrient dynamics in the medium as a function of intracellular variables and external variables like total number of cells.

The difficulty with this system is the heterogeneity of cells; each cell can have a slightly different behavior that the next one. Also cells can be in different parts of their cell cycle. It is possible to represent the different phases of cell cycle with a parameter, 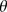, in the range of 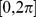. At 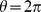 the cell divides and two new cells with phases 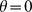 emerge. With this definition and assuming that all cells in the same cell cycle phase behave similar, each cell with index j can be known with its phase 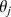.

There are different types of computational modeling techniques for solving such a problem. In some studies, different cells are referred with only one parameter like biomass and then it is connected to the population level dynamics with one equation, population-balance equation. These types of modeling do not consider the intracellular dynamics. Another class of models is cell ensemble modeling, where equation (1) holds for dynamics of intracellular quantities, but some randomness is added to the parameters in order to represent the deviation of cells from an average behavior. Therefore, there are ensembles of cells in different states in addition to eq. (2) to connect them to the common medium (for a review see: [57]). However, the number of cells that can be simulated in this way is limited, because the computational cost for these types of modeling is high and, therefore, it also puts a limitation on the amount of complexity in intracellular model.

In our model, we consider the intracellular dynamics for one type of cells, the type that is in majority after the synchronization. However, as we will show in this section, we make sure that the effect of non-synchronized (a minority of cells) also has been counted in the dynamics of the nutrient in the medium.

The experimental setup by which the data that we used was measured [52] is a population of diatom cells and a medium (artificial seawater). Moreover, prior to the measurements, there is a period of silicon starvation, in which cells have been kept in a silicon-free medium for 24 hours [52]. The reason for this preparation period, as we explained in introduction, is to synchronize cells as much as possible to create a framework for using the macroscopic scale, population-level quantities to infer cell-level dynamics. Therefore, the total amount of silicon consumption, after the start of the experiment and adding silicon, is due to consumption by all present diatoms in the population. However, it is known that only up to 80% of cells are synchronized in the best case [29]. In other words, not all the cells will stop their cell cycle in the main arrest point. For this reason, if we look at the cell population or cell density measurement during the observation time, for about 2 hours cell density remains almost constant but afterwards, it starts growing for the next 3 hours [52]. If the synchronicity were perfect we would expect cell density to remain constant until around the end and then a rapid growth to happen to make the population double in size, due to cell division.

We define 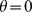 to be the phase where most of the cells after synchronization are located, which is somewhere in 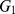 phase. Consider 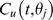 to be the total amount of silicon uptake by cell j at time t with the unit of 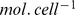, 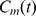 (equivalent to S in eq. (2)) to be nutrient (silicic acid) concentration in the medium at time t with the unit of 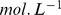
and 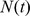 as cell density at time t with the unit of 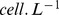
and 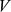 to be the total volume of the cell culture with the unit of 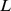. Thus the mass conservation law for nutrient amount is as follow,
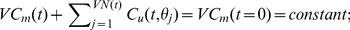
(3)


Assuming all the cells to have the same function of 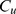 as the synchronized cells, then, 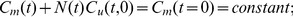
(4)


The time-derivative of eq. (4) results in, 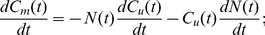
(5)


The right-hand side of equation (5) contains two terms. The first term is the summation of uptake rates of all cells in the culture, assuming that they were all synchronized. The second term, however, is representing the effect of non-synchronized cells; if number of cells stays constant during the observation time, the second term is equal to zero. Therefore, the nutrient in the medium decreases not only because of nutrient uptake by cells but also because of emerging new cells. Eq. (5) then will be connected to the intracellular dynamics of a synchronized cell. We can say that a typical synchronized cell gets information about other (non-synchronized) cells being divided, only through the common nutrient in the medium concentration. In the results section, we will show the role of each term in the whole amount of silicon consumption in our model and thus in the intracellular dynamics.

#### Single cell dynamics

There are different methods for modeling transportation through biological membranes. Based on the information derived from experiments, a specific kinetic pathway is inferred. Usually, for the sake of simplicity, only the chemical pathways representing the most important and relevant physiological events in a single cell are considered. The uptake rates of silicic acid in diatoms in most cases has been observed to have a saturable curve, which can be described with a Michaelis-Menten kinetics. In the case of exponentially growing diatom cultures and in short incubation times, the kinetics is reported to show a biphasic behavior [50]. However, in kinetics of uptake rates for a synchronized culture of cells, which have been in silicon starvation for a long period before the experiment, always has shown a saturable behavior (For more discussion see Text S1 from supplementary materials). Since we are using the data related to the synchronized cells, in our model, we use Michaelis-Menten enzyme kinetics for silicon transportation towards the cell and towards the SDV. We also use the enzyme kinetics for silica deposition as an event happening inside SDV.

Assuming that the uptake sites are located on the membrane only and nutrient species can freely diffuse, then we describe enzymatic processes by means of the following reactions: 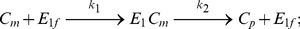
(6)

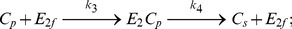
(7)

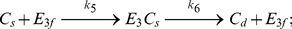
(8)where 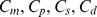 are the concentration of silicon in the four compartments medium, cytoplasm, SDV and deposited silica respectively. 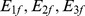 are concentration of free active sites of proteins (the first two, transporters and the third deposition-related) and 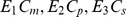 are the temporary occupied active sites in each time step. 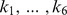 are equilibrium constants of transportation equations.

Based on immunoblots data, SIT protein levels change during the cell cycle. A minimum occurs in the S phase of the cell cycle when silica deposition is not taking place [44]. We assume that the total amount of enzymes is constant during the time for the first simulation. This is based on classical enzyme kinetics. For the second simulation, we define relative changes of total SITs as a function of time based on the relative level of protein changes coming from experimental data [44].

Therefore, equations (9–11) describe the conservation of the total number of proteins. In the second case, the right hand side of equation (9–10) is multiplied by a dimensionless function of time. 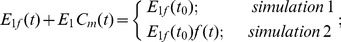
(9)

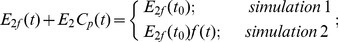
(10)

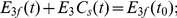
(11)


In eqs. 6–8 there are ten *state variables*, variables in equations which together determine state of the system, changing over time, but, considering the above constraints, only seven of them are independent from each other and thus seven variables are enough to describe the state of the system. Nevertheless, we will still use all of these equations in the model for the purpose of a better representation of enzyme kinetics. Some initial values of quantities involved in reactions are known, based on our choice of model setup and experimental data (timing of the experiment and starvation period) (See eq. (12) for details). However, there are some quantities where their initial values have to be considered as unknown parameters too. These are initial amount of free enzymes, for which, to the best of our knowledge, there is no quantitative measurement of the number of active proteins. There are, however, some studies that use the immunoblot of cell proteins to measure SIT proteins level during the cell cycle. This kind of experiment shows protein level changes over time, so it is a relative measure and not the absolute number [58], [44]. There is only an estimation of these numbers in literature that we have used them to choose the range of search space for these parameters in optimization simulation (see table 1).

**Table 1:** Parameters of the computational model.

Parameter	Description	Dimension	Value	Reference
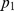	Half-saturation constant1	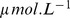	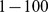	[50]
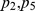	Maximum rate constant 1 and 2	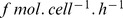	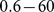	[50]
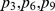	Initial number of active sites 1,2 and 3	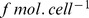	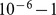	[50], [45]
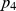	Half-saturation constant 2	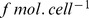	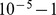	[50]
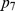	Half-saturation constant 3	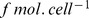	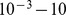	[45]
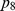	Maximum rate constant 3	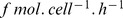	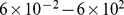	[45]
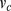	Cell volume	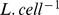	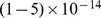	Our data
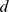	Cell diameter	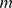	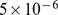	Our data
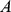	Cell surface	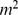	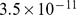	Our data
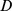	Diffusion coefficient of silicic acid in water	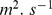	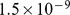	[41]

The values for the range of parameters for the optimization search are approximate values, based on experimental measurements.

In enzyme kinetics, usually the kinetic parameters of chemical reactions are introduced in terms of half-saturation and maximum rates. For a better comparison with literature we changed the kinetic parameters of the model (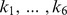) to this type of parameters (vector 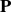 in eq. 12). These parameter changes come from Michaelis-Menten calculations. Moreover, we added 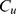, uptake by one cell, to the list of states variable to connect equations of individual and population level (vector 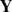 in eq. 12). All of the state variables are in the units of 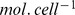
except 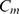, which has the unit of 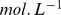. Finally, we define the parameters vector, state variables vector and their initial values, immediately after silicon starvation, for solving ODEs as shown in eq. (12).
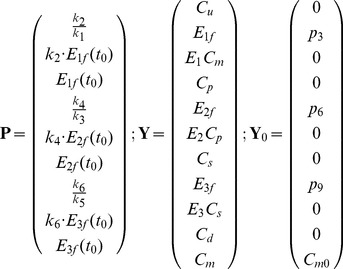
(12)


Using the law of the mass action and equation (5) derived from mass conservation, model system of equations is complete. It is provided in supplementary material Text S2, equations (S2–2)–(S2–12).

### Problem of parameter estimation– Inverse problem

#### Formulation of the optimization problem

To understand sub-cellular processes, computational modeling, based on knowledge of the cell cycle and experimental data mostly at the population-level, is a strong and necessary tool. Using a mathematical model to produce the results comparable with those obtained from an experiment is called the *forward problem*. Equations (S2–2 to S2–12), assuming availability of all parameters, describe a forward problem. However, when it comes to real cases, most of the models for intracellular dynamics contain several unknown parameters. This is usually unavoidable because the measurement of transport rates during the observation time and for all compartments in vivo is not feasible and any data that can estimate them might contain a large error. Such a problem often appears in biological systems. To overcome this we need to design a parameter estimation or optimization problem i.e. to find the best set of parameters that makes the model output fit to available experimental data as closely as possible. This is called the *inverse problem*. The inverse problem is not always solvable and it critically depends on the mathematical model, significance and accuracy of data [59]–[61]; A model that conveys too much complexity compared to the available data, insufficient data or noisy data can result in a failure in solving the optimization problem. The entire method is an iterative process meaning that the designed model and its optimized solutions can predict some unavailable data and the result will shed light on our understanding of how those mechanisms work, in order to predict a more suitable model and to provide more realistic data estimations.

The main task in optimization is to minimize a problem-dependent objective function or cost function. In the general form, the objective function is made of two terms as follows, 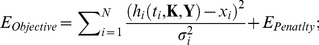
(13)where 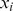
s are the observed experimental data and 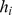 is a function of model quantities used to calculate the equivalent measure. 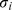 is the standard deviation of experimental errors in the data. The first term is based on *weighted least square* method which is the most common method for building objective function [62]. The second term in equation (13) is the *penalty term*. When there is a constrained problem, the penalty method helps to turn the constrained problem to an unconstrained problem and in this way, we are able to use the successful optimization algorithms for unconstrained problem to solve the constrained problem [63].

The criterion for accepting the objective function (objective tolerance) is defined based on the amount of error in the input data, which is our benchmark for fitting. The range of parameters that result in this range of the objective function is called the acceptance interval. If any changes in parameters can produce a value for 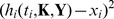 that is smaller than 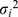, those changes are not meaningful anymore. Also any attempt to find a smaller 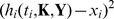, will results in overfitting to the data and will add the experimental data noise to the numerical model; something that should be avoided as much as possible.

Often, the input information is insufficient to solve the inverse problem or, in other words, the parameters cannot be estimated. The input information consists of the model structure, the data used for fitting and the constraints on the model. We will show in results section that considering the choice of data, placing some constraints via a penalty function is necessary for our compartmental model. Equation 14 is the penalty function that we have used in our model.
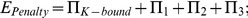
(14)


Some of the constrains we aim to impose on our model, limit the search space of parameters (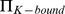) and this is of great importance. When we have some pre-knowledge about the range of the parameters from literature, it is necessary to apply them in order to reduce the computational time of the solution convergence and also likely increase the quality of the solutions themselves. Another group of constraints comes from our knowledge of the state variables. We have imposed these types of constraints through functions: 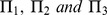. One important constraint in enzyme kinetics is that the amount of enzymes is much lower than the total amount of substrate and product (because enzymes are not consumed during the reactions). We represent this constraint mathematically as equation 15. 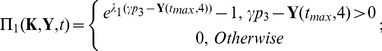
(15)


If the constraint in Eq. (15) is violated, then a penalty term is added to the cost function and that set of parameters is pushed to be far from the minimum cost and therefore less favorable as the final solution. The penalty function is by definition, continuous, as it is in eq. (15). 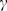 is a parameter that imposes the strength of inequality. A bigger 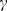 brings a stronger constraint. 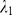 is called the regulatory factor or penalty coefficient. It is a tuning parameter used to make the “penalized unconstrained problem” as similar as possible to the original constrained problem. We have chosen this value such that it adds a penalty value comparable to the first term of the objective function, once the constraint is violated. The value for 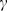 has been chosen to be relatively small (5 in most experiments) in order avoid a very strong constraint and to allow a bigger range of acceptance for parameters. For the second and the third enzymes, similar expressions provide the definition of the last two terms in the penalty function (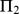
and 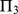).

#### Choosing experimental data

There have been several experimental studies on *T. pseudonana* silicon uptake rates and population growth. Table 2 shows a summary of some of the studies, where each of them has slight or major differences in setups and initial conditions. We presented these conditions together with some of their measurements in table 2.

**Table 2:** The relevant experimental measurements on diatom *T. pseudonana* measurements.

Growth Condition	Measurements		Reference
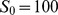 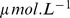	Population growth = 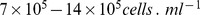		
SD = 24 h	Si decrease in environment during one cell cycle = 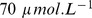		[52]
T = 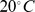			
Light-Dark: 14 h–10 h			
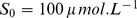	Uptake rates at:		
SD = 5–10 min	T = 2 min –33 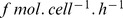	T = 10 min –17 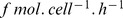	[50]
T = 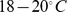	T = 30 min – 4 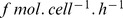	T = 1 h – 6 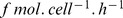	
Continuous light	T = 2 h – 4 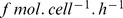	T = 3 h – 4 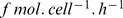	
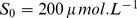	SIT protein level changes during cell cycle: 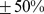		
SD = 24 h	Uptake amount = 0–40 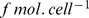		[44]
T = 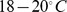			
Continuous light			
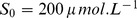	Synchronized percentage = 60–80%		
SD = 24 h	Uptake amount = 0–80 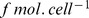		[29]
T = 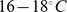	Approximate amount of silicon in each valve: 20 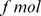		
Continuous light	Final silicon pool = 15 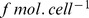		

The condition of the experiment: SD: starvation duration, T: temperature, 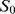: initial concentration of silicic acid after the starvation. The measurement values in this table are approximate.

The data that we have used for parameter estimation is the temporal changes in concentration of silicic acid in environment (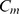) together with the population of diatoms (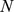) [52], from which we then find the best parameters in our compartmental model to fit for the measured values. Moreover, we include the one data point from the deposited amount of silica (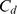), coming from the data on the average amount of silica in diatoms walls, which is approximately 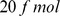 in each daughter cell of *T. pseudonana*. Therefore, this is the value that we consider, with a big error (at least 5%), for the final amount of 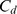 or 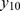.

#### Solving the optimization problem

There are many different numerical methods to find optimized parameters. Generally speaking, the methods are considered to be local or global. The first class of methods relies on local search algorithms. They are usually fast, but as the name suggests, they find local optima and they do not have a mechanism to escape that optima in order to find the lowest objective function over the entire search space of parameters. Usually the landscape of the objective function is unknown; therefore, there is often an essential need for a second class of optimization methods, which use global search algorithms. These types of algorithms are usually computationally expensive. There are a number of different global optimization methods, many of which have been applied to problems with biochemical pathways [64],[65]. For example, one method uses the so-called kinetic theory for active particles, where it can also reduce the number of parameters and therefore make the solution of the inverse problem more accurate [66],[67]. We have used scatter search, which is a population-based metaheuristic algorithm for global search. It shares many features with evolutionary algorithms, such as like genetic algorithms in principle [68],[69]. We combined the scatter search with a local search algorithm and a penalty function to achieve a good rate of convergence and a good set of solutions.

#### Parameter identifiability, sensitivity and perturbation analysis

**Figure 4:**
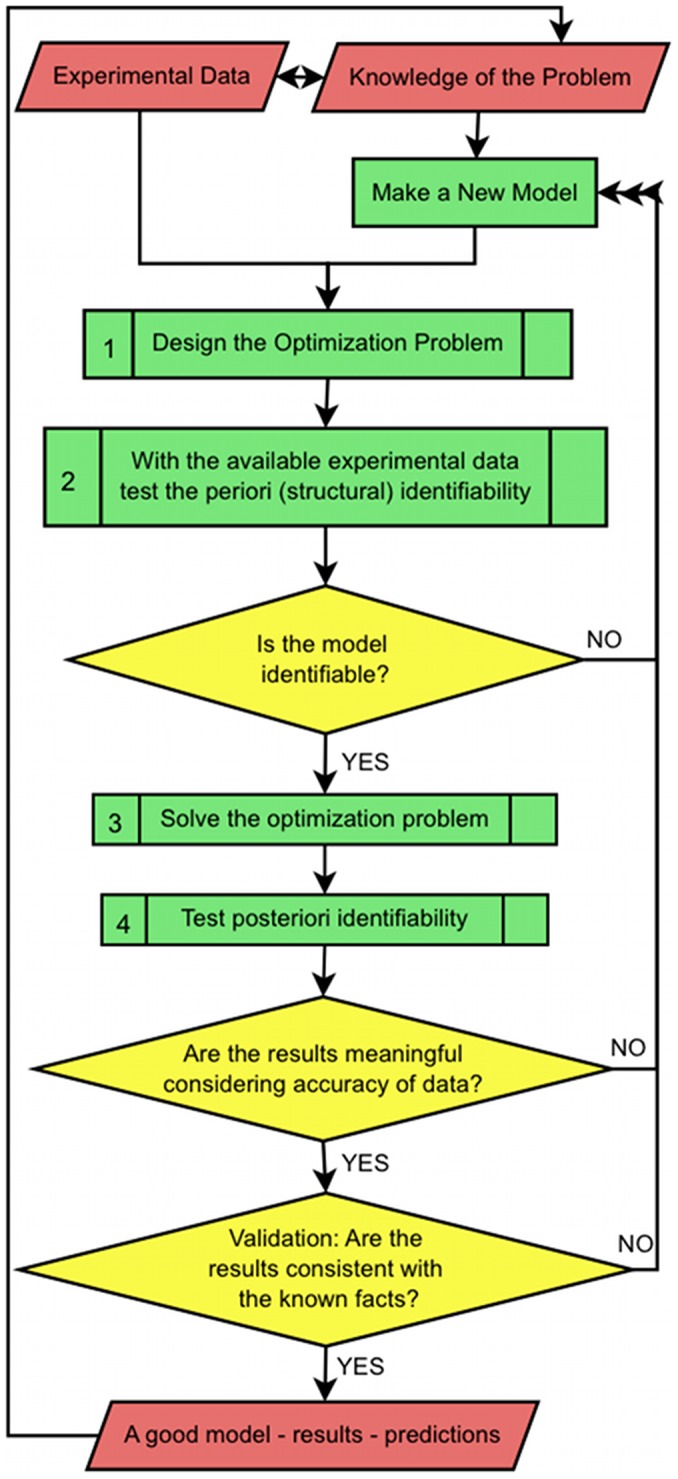
A flowchart for finding solutions to the inverse (optimization) problem. After designing an optimization problem, the first step is to check structural identifiability of parameters: to test if the inverse problem is solvable assuming that data are accurate and significant. After that the optimization problem should be solved (globally or locally) and at the last step posteriori analysis should be applied to ensure the results are meaningful and the model is robust. In the end, if the model can be validated, it can predict the mechanisms in the system, which, in turn, can provide a better model and, therefore, a better understanding of the system.

Designing a model and some experimental data to use as fitting criteria is only one part of the procedure for finding the valid unknown parameters and building a meaningful model. In fact, it usually does not happen that the optimized parameters found from the simulation are immediately the only and the correct solutions. For instance, the inverse problem often does not have a unique set of solutions [59]. Therefore, one should perform some pre and post analysis on the model and optimized solutions. Figure 4 shows a flowchart of the procedure towards finding best solutions. After defining the optimization setup, a priori or structural parameter identifiability should be tested. This type of identifiability analysis answers the question that considering error-free and continuous (observable in any desired time point) experimental data, whether it is possible to identify unknown parameters or, in the other words, whether or not parameters are locally or globally identifiable. In practice, sometimes, especially for large nonlinear system of equations, it is not easy or sometimes not possible to perform structural identifiability. But if it is a possibility it can save a large amount of time and effort because it is performed before solving the optimization problem and it can help to modify the model or the choice of data sets.

There have been many mathematical studies which address the question of structural identifiability [70],[71]. We tested our model's identifiability with GenSSI, which is a software system available for structural identifiability and is based on the generating series approach [72],[73]. The result of this analysis was that applying it to our model and having data on 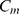 and 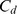 only, the model parameters are not globally identifiable. To handle this problem, we add extra information that we know from enzyme kinetics as constraints of the model. We used the penalty function approach to impose these constraints.

The results of our optimization problem support the analysis came from GenSSI on the idea that without a penalty function, our model parameters are not globally identifiable. We did a test for two cases: The objective function; (1) without any penalty term, and (2) with penalty terms presented in eq. 14 and then compared the scatter plots of parameters and all state variables in acceptable objective function range (acceptance interval). We show this comparison in the Results section. This comparison shows that in case 1, parameters are more scattered and the state variables are less determined compared to case 2, where we have applied penalty terms to the objective function.

In second step, optimization problem should be solved using an appropriate algorithm in order to find the unknown model parameters. The last step, which is usually as important as the second step, is practical or posteriori identifiability. It is common in practice that given the amount of noise in experimental data, there are several solutions for an optimization problem, or the model is not robust around some of optimized parameter values. Posteriori identifiability concerns this type of question [59]. The existence of noise in data leads to uncertainty in fitting criteria (cost function) and therefore to uncertainty in parameter values. To quantify posteriori identifiability we use sensitivity and perturbation analysis. Sensitivity is a measure of how big the changes are in one variable when a certain amount of change in parameters is imposed. The preferred situation would be that this measure is reasonably small.

Mathematically, the sensitivity of state variables to the change in parameter values is described by the sensitivity matrix, defined as,
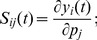
(16)


We will only calculate sensitivity of states that are observables of the model.

The normalized sensitivity, which is a better measure for comparing parameters, is as follow,
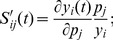
(17)


To calculate normalized sensitivity, either we can use the discrete differentiation of eq. (17) or we can use its derivative as an addition to the ODE system of the model. 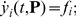
(18)

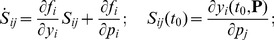
(19)


Together they make a new set of ODEs, which should be solved again.

Another important evaluation for the solutions is perturbation analysis. By adding some perturbation to the parameters around their optimum value, one can analyze how much it perturbs the system; both the cost function and all the variables of the system. To conclude that a model is stable and robust, we need a model that does not amplify small perturbations, which is also the case in any real stable biosystem.

## Results

**Figure 5:**
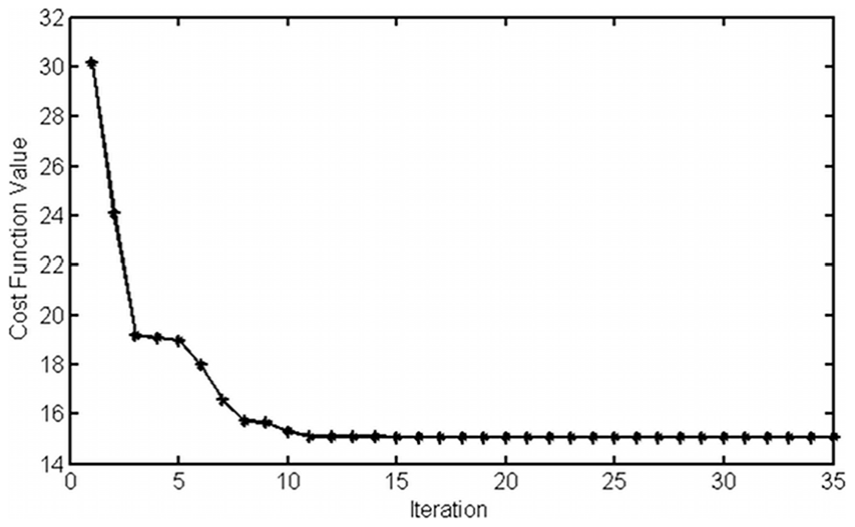
Objective function value. Best objective function during searching for optimized solution until the algorithm cannot find a better value for a long time. The simulation time was around 12 hours.

Using scatter search simulation in the search space of parameter values, the minimum for the objective function has been achieved. The search space that we have used here is listed in table 1. The approximate values for proteins came from literature, which are mostly based on macroscopic properties of enzyme kinetics. Figure 5 shows the best values for the objective function. The algorithm stops searching when the acceptance criteria is achieved, which is when the algorithm has not found any better solution for a long time and the objective function is close to the optimum value. Since we have considered a weighted objective function for 10 data points (eq. 13) the best meaningful value for objective function is 10 and if the search algorithm arrives at values less than 10, all of those solutions are acceptable and there is no preference among them, in order to avoid overfitting problem. In the simulation shown here, the objective function is around 15. So, with the considered search space for parameters, this model is able to closely reproduce the experimental data with a relatively small fitting violation. The solution does not change by repeating the scatter search or by changing the initial point (results are not shown).

**Figure 6:**
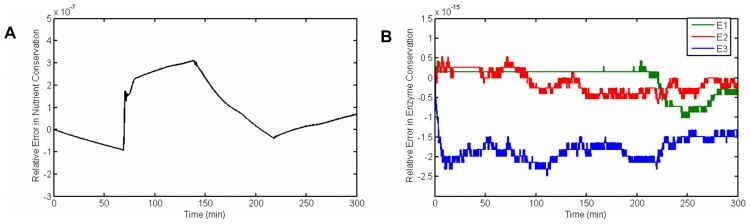
Relative error in conservation rules. (A) Conservation of nutrient (eq. 2), (B) Conservation of enzymes (eqs. 9–11)

We also make sure that the model equations hold the conservation law for nutrients and enzymes. Figure 6A shows that the relative error in conservation of silicon is of the order of 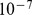 and Figure 6B shows the relative error of total protein conservation is 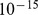, both of which show a very good accuracy of the numerical solvers.

**Figure 7:**
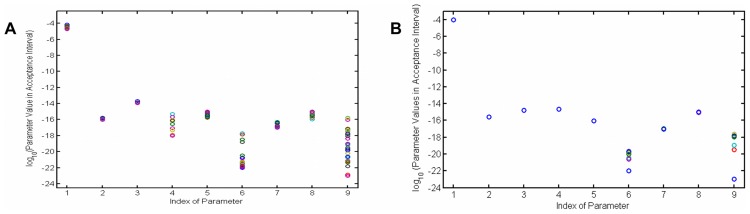
Scatter plot of accepted parameters. (A) Parameter values in the acceptance range of objective function for case 1, when there is no penalty term in the model. Most of parameters are scattered in this case, which means that there are many solutions to the optimization problem. (B) Parameter values in acceptance range of objective function for case 2, when penalty terms added to the objective function. In this case, besides 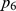 and 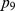, most of the parameter values are not scattered much.

**Figure 8:**
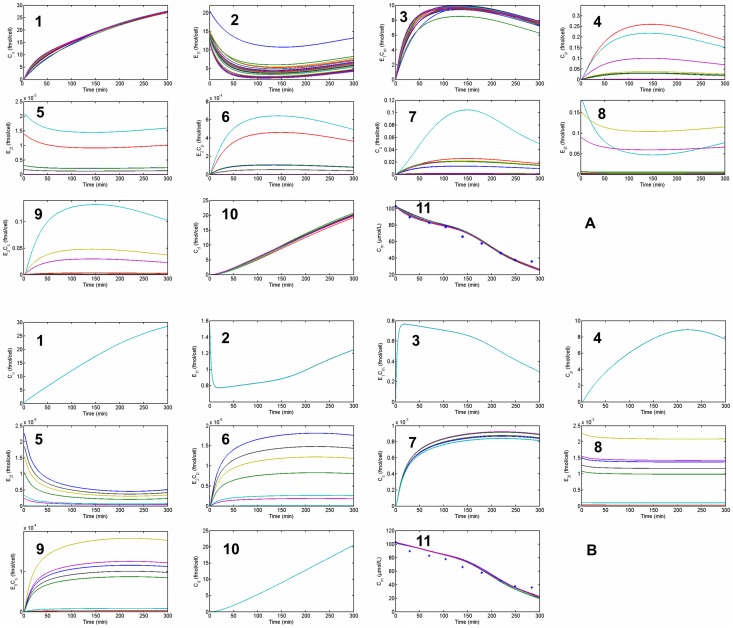
Model output of forward problem resulted from different solutions of the inverse problem. The temporal dynamics of 11 variables of the model in (A) case 1 with no penalty term and (B) case 2 with penalty terms. Even though there are different curves for variables of both cases, using penalty term made the silicon dynamics (1,4,7,10,11) much more unique and identified.

The problem has several solutions all in the same range of the minimum cost function value. These accepted parameters are shown in a scatter plot of parameters in Figure 7. To clarify the role of penalty terms, two cases of simulation have been performed, as discussed in previous section. In the first case, no penalty function imposed to the model (Figure 7A) and in the second one penalty terms are added to cost function (Figure 7B). From the results it is clear that with no penalty, parameter values are mostly scattered, but applying the penalty, besides 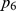 and 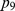, they mostly form one cluster and are not significantly scattered. This means that in the second case, most parameters are very well identified. Moreover, with the optimized parameters, we return to the forward problem and calculate temporal dynamics of intercellular quantities. Applying an accepted set of parameters, we test the variations in the model variables to make sure there is not a different behavior when changing from one solution to another. Figure 8 shows the concentration dynamics of silicon and enzymes in all cell compartments with and without the penalty term. With no penalty, the dynamics of most variables changes for different set of parameters, however, after applying penalty, although there are some variations in few of the variables, still there is the same dynamics for concentrations, especially concentration of silicon in different compartments.

**Figure 9:**
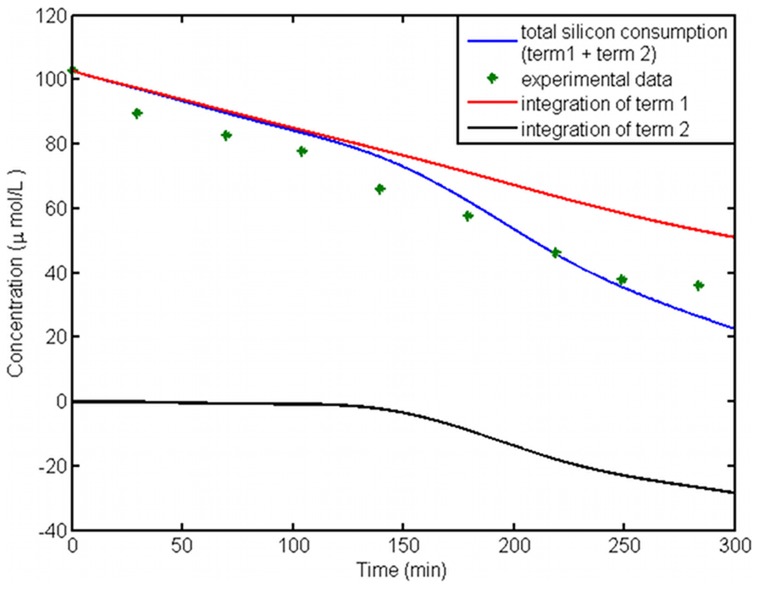
Silicon concentration in the medium – fitting to experimental data. Data from the experiment and model output of silicon concentration in the culture medium is shown in points and a blue curve. This curve depicts the total silicon consumption by all cells. The red curve is the integration of uptake rate coming from term 1 in equation 5. After 150 minutes, the difference between this value and the total silicon consumption becomes big as a result of non-synchronized cell culture. Adding the black curve, integral of term 2, compensates for this difference.

In Figure 9, experimental values for silicon concentration in the medium together with the model output is shown. The fitting to data, considering experimental error is appropriate. This figure also shows the two curves illustrating the effect of the two terms in eq. (5), which was discussed in earlier. We calculated the integral of each term, with a trapezoidal method, to calculate their contribution in silicon consumption. It appears that term 2 in the beginning does not have a significant effect and term 1 has the major role. However, the absolute value of term 2 starts to grow through time and reaches a considerable value. This effect is consistent with the behavior of the diatom cell cycle in a bulk. First, cells are not dividing, but instead up-taking silica with the highest rate in order to build the silica valves for daughter cells. Even though, some cells divide earlier than others, they are only in the beginning of girdle band growth and the uptake rate is not very high and therefore, the first term becomes dominant. By the end of the experiment's duration, the second term has a considerable role, which shows that it has to be included in the calculations of synchronized cell cultures.

To this point we concluded to the solution of compartmental dynamics based on our mathematical model. The fact that parameters are mostly not scattered is a good sign. However, if a model is very sensitive to certain parameters, even a small change can possibly affect the dynamics significantly. Therefore, in order to check the robustness of our model, we first performed sensitivity analysis on solutions and then measured the effect more clearly by means of perturbation analysis.

**Figure 10:**
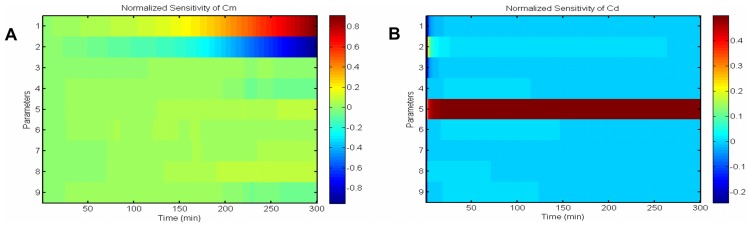
Sensitivity analysis. Parameter sensitivity matrix around the solution, for variables that have been used in fitting procedure. (A) Normalized sensitivity of silicon in the environment, 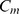, shows it is most sensitive to changes in 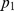 and 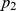. (B) Normalized sensitivity of deposited silica, 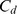, shows it is most sensitive to the changes of 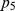.

**Figure 11:**
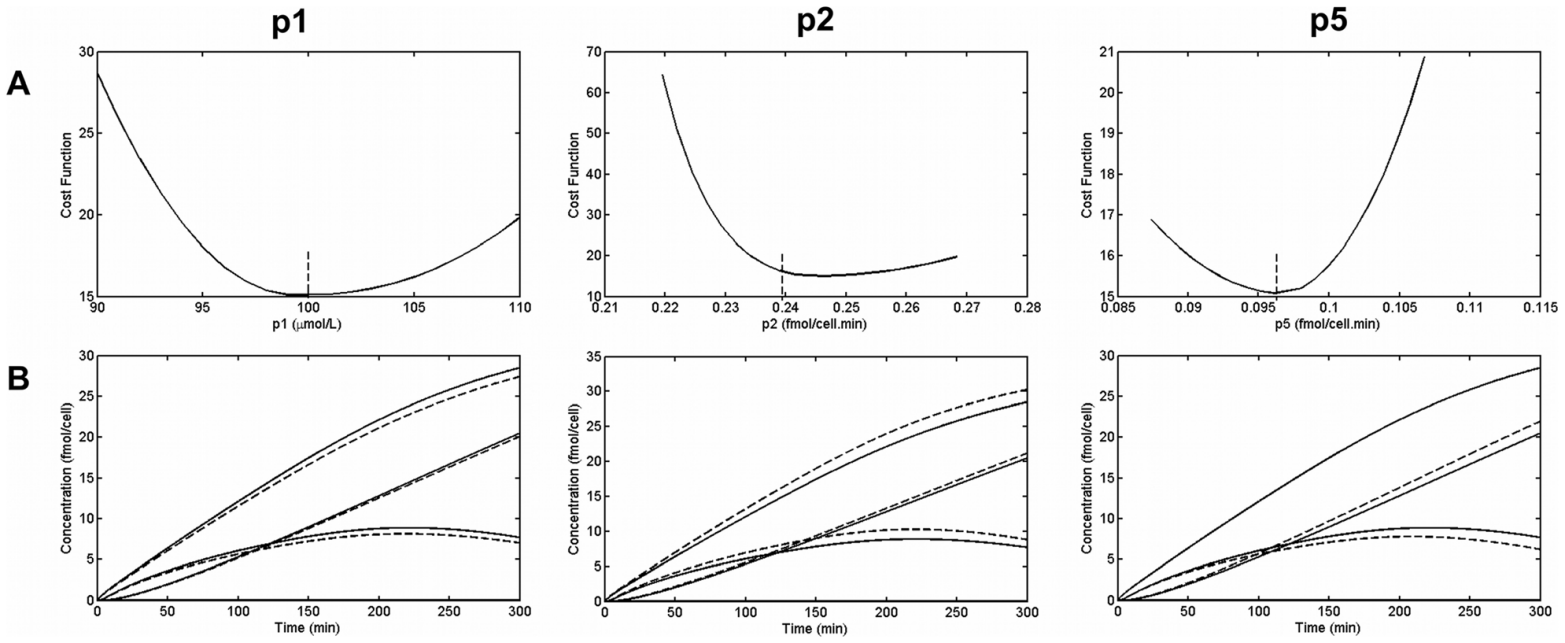
Perturbation analysis. Changes in, (A) objective function and (B) intracellular dynamics of nutrients, due to perturbation of system by 10% change in parameters 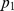, 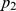 and 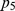 (solid line: original quantities - dotted lines: perturbed quantities).

Figure 10 shows the normalized sensitivity of observables by changing parameters. Since we have only used the data from medium concentration and deposited silica, we investigate their sensitivity. 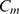 is most sensitive to parameters 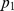 and 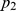, especially at the end of the experiment where 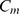
decrease has a smaller slope. 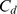 shows greatest sensitivity to 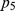. To bring an understanding of the amount of change that can be produced by these important parameters, we have again performed perturbation analysis. In Figure 11 the results of parameter perturbation are shown in terms of objective function and temporal dynamics of concentrations. We can see that with a relatively large change in the parameters (10% perturbation), the behavior of solutions remains the same. For a nonlinear system, this is a reasonable amount of change and it means that our model is robust and stable.

**Figure 12:**
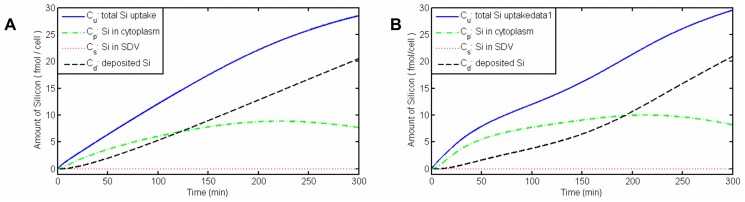
Dynamics of silicon concentrations in different compartments. (A) Silicon transport with the assumption of a constant amount of enzymes in all compartments. (B) Silicon transport considering a flux of proteins for SITs during the cell cycle.

Figure 12A shows the silicon content dynamics in different compartments. From the total amount of silicon uptake by cell, the biggest amount becomes in the form of deposited silica, as we desired. However, there is some silicon in cytoplasm or, as it's called, the silicon pool. The concentration of non-deposited (dissolved) silicon in SDV (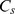) is very low. This makes sense considering very small volume of SDV and also the fact that SDV grows together with silica valves and thus leaving only a small portion of the compartment for non-deposited silicon. As we discussed previously, cells control the uptake and transport of silicon through changing the expression level of SITs. To test the effect of these changes on uptake rates by the cell, we have added a flux term to equations S2–3 and S2–6 for the amount of free enzymes (SITs) during time and again solved the inverse problem. We have chosen this flux as a function of time based on changes in protein level from the experiments [44].

**Figure 13:**
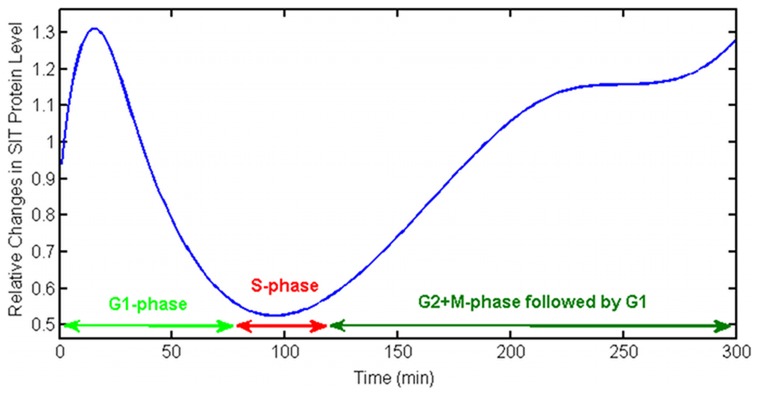
Protein flux used in the model. The relative changes in SIT protein expression level during the cell cycle of diatoms that has been applied to the model. This curve is inferred using the data from the experiment [44 - Figure 3]. SITs are least expressed during S-phase when silicon deposition is almost stopped.

The relative change of SITs that we applied to our model is shown in Figure 13. This function is chosen based on experimental data, but it only shows the behavior of the SITs expression level. The main idea is that during the S-phase, while silicon deposition is minimum, the amount of proteins is also at its minimum. Figure 12B is the result of imposing the protein flux on the silicon amount in different compartments. It shows that even though the total dynamics are more or less the same, but there are some differences. For example, in experimental measurements it seems that silicon deposition happens in a low rate immediately after adding silicon to the starved cells. This might be because of silicon storage in cytoplasm in the beginning [29]. The 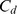
dynamics in Figure 12B is closer to this behavior than 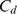 in Figure 12A.

**Figure 14:**
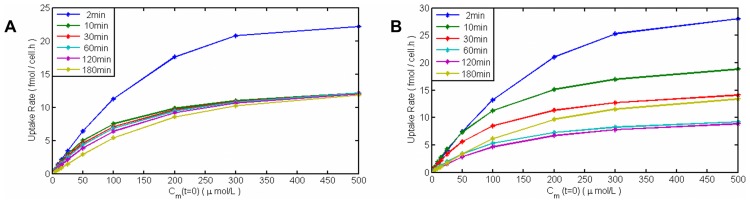
Silicon uptake rates of diatoms versus silicic acid concentration in medium in different time steps. (A) Constant amount of total enzyme. (B) Considering changes in total amount of SIT enzymes. The uptake rates have saturated forms in high concentrations of dissolved silicon in water. 2 minutes after adding silicon to the starved cells, the uptake rate is very high (surge uptake). By passing time the uptake rate has a big drop in value. In (B) this drop accrues slower and the rate increases again during valve formation.

An important measure in nutrient uptake is the uptake rate. In Figure 14A we have shown uptake rates versus medium concentration, measured by imposing different initial values for silicic acid concentration in the medium or cell culture (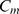) and then the calculating the uptake rates at different time points. The uptake rate-silicic acid concentration curve is a saturated curve as expected. Uptake rates in the first time step, after 2 minutes, are much higher than later uptake rates. This can be a result of the initial condition of the model, zero value of silicon inside the cell, which we assumed based on the fact that cells have been kept in silicon starvation for a long time (24 hours) prior to the measurement. Also this big drop shows the nonlinear nature of the system. This sudden decrease has been seen in experiments on diatoms. It is called surge-uptake. Also experiments suggest that in low concentrations of silicic acid, uptake is almost linear and thus it is referred to as externally controlled (cells uptake as fast as the diffusion of external nutrient allows them). However, in high concentrations of silicic acid, the uptake rate reached its maximum value and, therefore, it is called internally controlled uptake. Interestingly, this model is able to produce all three phases of uptake: (surge uptake, internally controlled and externally controlled), with only the enzyme kinetics and considering cell compartments and without any other assumptions on the presence of different phases of uptake in diatom cells. Figure 14B is the same measure for the case when we applied changes to the total amount of SITs. Compared to 14A, the decrease in uptake rates accrues slower, which is closer to some experimental data of uptake rates in different incubation times [50]. Moreover, the uptake rate increases again at the time of starting valve formation, when the cell needs a high amount of silicon.

## Discussion

Studying intracellular processes is a difficult task, largely because of the difficulties of in-vivo measurements at such small scales. As a consequence of this, the modeling approach is a good candidate for understanding the different mechanisms and temporal behavior, such as nutrient uptake and transportation of materials. We have introduced a multi-compartmental model for silicon transport and synthesis in diatoms. This is the first nonlinear compartmental model designed for this purpose. The model uses experimental population-level (macroscopic) data and therefore an accurate equation for the connection of macroscopic data to sub-cellular data has been derived. This equation accounts for the effect of a portion of cells that are not synchronized in the cell culture. To estimate the free parameters of the model, we have solved the inverse problem of parameter estimation, which includes the constraints related to enzyme kinetics via the penalty method. The use of constraints together with sensitivity and perturbation analysis makes it possible to infer the sub-cellular dynamics even with sparse and noisy data. Taking into account the amount of silicon (mol) in the model in order to simplify different polymerization and other reactions in cells, the model is still able to produce the transport and the dynamics of nutrients with a good agreement to experimentally obtained evidence. For example, the equations of the model can generate different regimes of uptake, namely: surge, externally-controlled and internally-controlled.

This model constitutes a framework by which to study cell compartments and to infer dynamics based on the known facts and data. More importantly, it brings with it the ability to manipulate cell processes such as uptake rates and protein regulation, which are normally difficult to manipulate experimentally. As an example, we have imposed changes in the total amount of SITs through time; a mechanism for cells to control the uptake and transport of materials. We have observed that applying a protein flux results in more realistic silicon dynamics and uptake rates, and can postpone the start of silica deposition in favor of silicon storage in the silicon pool.

The model presented here can be generalized to other chemical pathways by changing only the system of ODEs, which lie at the core of the model, and then applying the same procedure. One interesting area in this framework would be to introduce changes in the expression level of enzymes during time as a function of the internal and external changes, and try to connect the cell cycle and silicon cycle of diatoms together. Another interesting addition to this type of model would be to consider the statistical distribution of individual cells' dynamics in the culture, which will introduce more complexities. This can be achieved by applying individual-based modeling, or with an ensemble based modeling to fulfill the role of cells in different phases.

## Supporting Information

Figure S1
**Two types of diffusion flux in nutrients dynamics.** 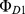 is the flux of diffusion through membrane. 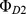
is the diffusion flux that controls concentrations of substrate and product near the membrane, where enzymatic reaction occurs.(TIF)

Figure S2 **Comparison of the rates of diffusion-mediated uptake with no-diffusion uptake.** (A) and (B) uptake rates for two observed values of MM coefficients. The curves are almost the same. (C) The same as B with diffusion constant one order of magnitude smaller. The effect of diffusion is still negligible.
(TIF)

Text S1 **The effect of pre-starvation and diffusion on uptake rates dynamics.**
(DOCX)

Text S2 **Intracellular dynamics system of equations.**
(DOCX)
