# Supplementary figures and images for: Understanding the Sub-Cellular Dynamics of Silicon Transportation and Synthesis in Diatoms Using Population-Level Data and Computational Optimization
Source: PLoS Comput Biol. 2014 Jun 19;10(6):e1003687. doi: 10.1371/journal.pcbi.1003687 (PMC4063665; doi:10.1371/journal.pcbi.1003687)

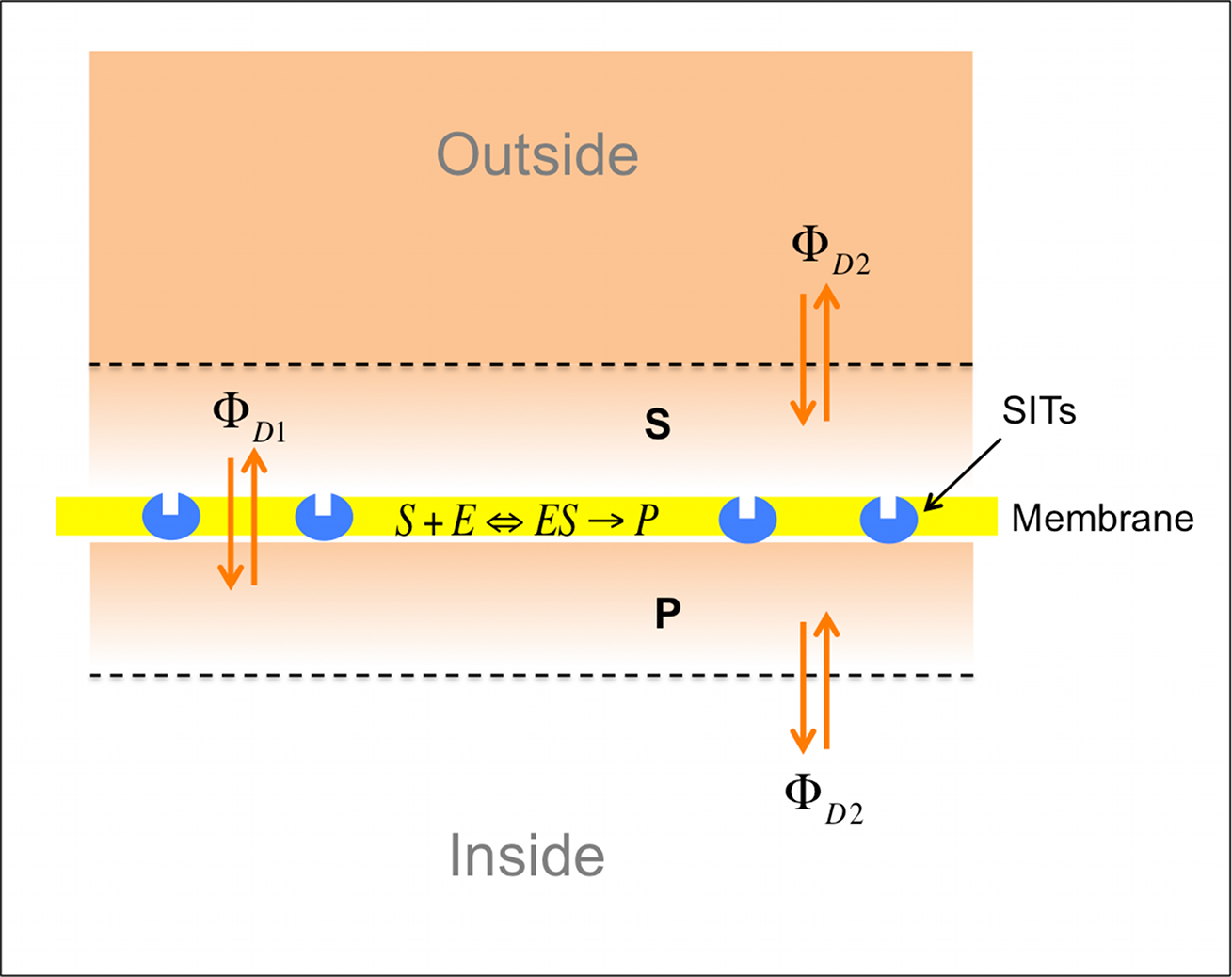

Supplement: Figure S1 — Two types of diffusion flux in nutrients dynamics. is the flux of diffusion through membrane. is the diffusion flux that controls concentrations of substrate and product near the membrane, where enzymatic reaction occurs. (TIF) [file pcbi.1003687.s001.tif]

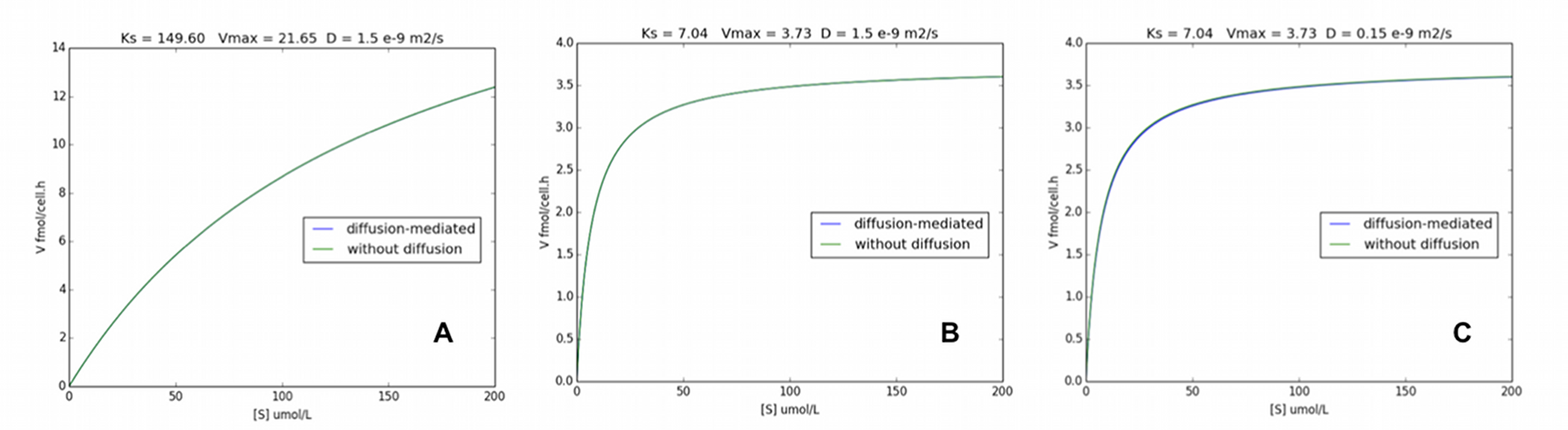

Supplement: Figure S2 — Comparison of the rates of diffusion-mediated uptake with no-diffusion uptake. (A) and (B) uptake rates for two observed values of MM coefficients. The curves are almost the same. (C) The same as B with diffusion constant one order of magnitude smaller. The effect of diffusion is still negligible. (TIF) [file pcbi.1003687.s002.tif]
