# Supplementary material for: Understanding the Sub-Cellular Dynamics of Silicon Transportation and Synthesis in Diatoms Using Population-Level Data and Computational Optimization
Source: PLoS Comput Biol. 2014 Jun 19;10(6):e1003687. doi: 10.1371/journal.pcbi.1003687 (PMC4063665; doi:10.1371/journal.pcbi.1003687)
Supplement: Text S1 — The effect of pre-starvation and diffusion on uptake rates dynamics. (DOCX) [file pcbi.1003687.s003.docx]

**Supplementary Materials: Text S1**

The effect of pre-starvation and diffusion on uptake rates dynamics

Effect of pre-starvation

The kinetics of silicic acid uptake by diatoms in most cases can be described with a Michaeles-Menten kinetic. However, in the case of exponentially growing diatom cultures and in short incubation times, the kinetics is reported to show a biphasic behavior [50].

In [50], two different cases have been discussed. **Case 1**, when diatoms were kept for long term (24 hours) under silicon starvation condition before the measurements and **Case 2**, when diatoms were pre-starved only shortly (5 to 10 minutes). Then in each case the uptake rates were measured in different incubation times. They reported that shortly after start of the experiment (after adding silicic acid) at t=2 min, the uptake rate vs. initial concentration of silicic acid in the medium, in case 1, showed a saturable form (meaning it reaches a maximum and therefore, it can be described by MM kinetics), while in case 2, this curve was in general non-saturable (saturable in low concentrations, but linear at higher concentrations). The later behavior is called biphasic uptake. In longer incubation times (t=30 min, t=1h, etc.) both cases showed a saturable kinetics. Thamatrakoln et. al. suggested that the linear part of the curve in that case could be a result of control by capacity of the silicon pool via binding to a hypothetical binding component [50]. Moreover, they suggested that the flux of silicon in linear part is diffusional. In the next section we will discuss about the possibility of diffusion across the membrane as a method for nutrient supply. The data that we have used in the current study is driven from an experiment in which a 24h pre-starvation was performed [52]. Therefore, the use of saturable MM kinetics in our model, even for high concentrations and short incubation times, is still consistent with data from other studies too [e.g. 50].

In terms of the model parameters, the role of pre-starvation of cells, defines the initial condition of the model. If there was not a long pre-starvation, the initial condition and therefore, the resultant kinetics would be different. For example, for the long pre-starved cells, the concentration in silicon pool at t=0 is assumed to be zero in our model or all the enzymes are assumed to have free uptake sites. Moreover, the 24h pre-starvation causes up to 80% of the cells to be synchronized in their cell cycles and therefore, cell density in the medium will remain almost constant for the first few hours [29]. In contrast, if cells were not kept in long starvation (like case 2 of [50]) cells will be randomly distributed in different parts of their cell cycle (for instance, there would be a significant number of cells that divide immediately) and this would make the uptake rates of cells very different from each other. As a result, one cell could not be a good representative of all the population. This is in fact one reason for performing long pre-starvation. Modeling a non-synchronized system would need a more complicated statistical simulation, where cells in different phases of cell cycles can be taken into account and the changes of silicic acid in the environment would be due to different cells behavior in different times.

In summary, the current model is related to the dynamics of pre-starved and therefore, synchronized diatoms. This specifies their initial condition, their population synchronicity and their [possibly] specific choice of types and amount of SITs expression. Moreover, we will use the ordinary differential equations (text S2) of enzyme kinetics and not the closed-form equation of rate derived by MM kinetics to reduce the approximations as much as possible.

Effect of diffusion

In this section we will discuss the effect of the mass diffusion of the silicic acid on the dynamics of uptake and transportation.


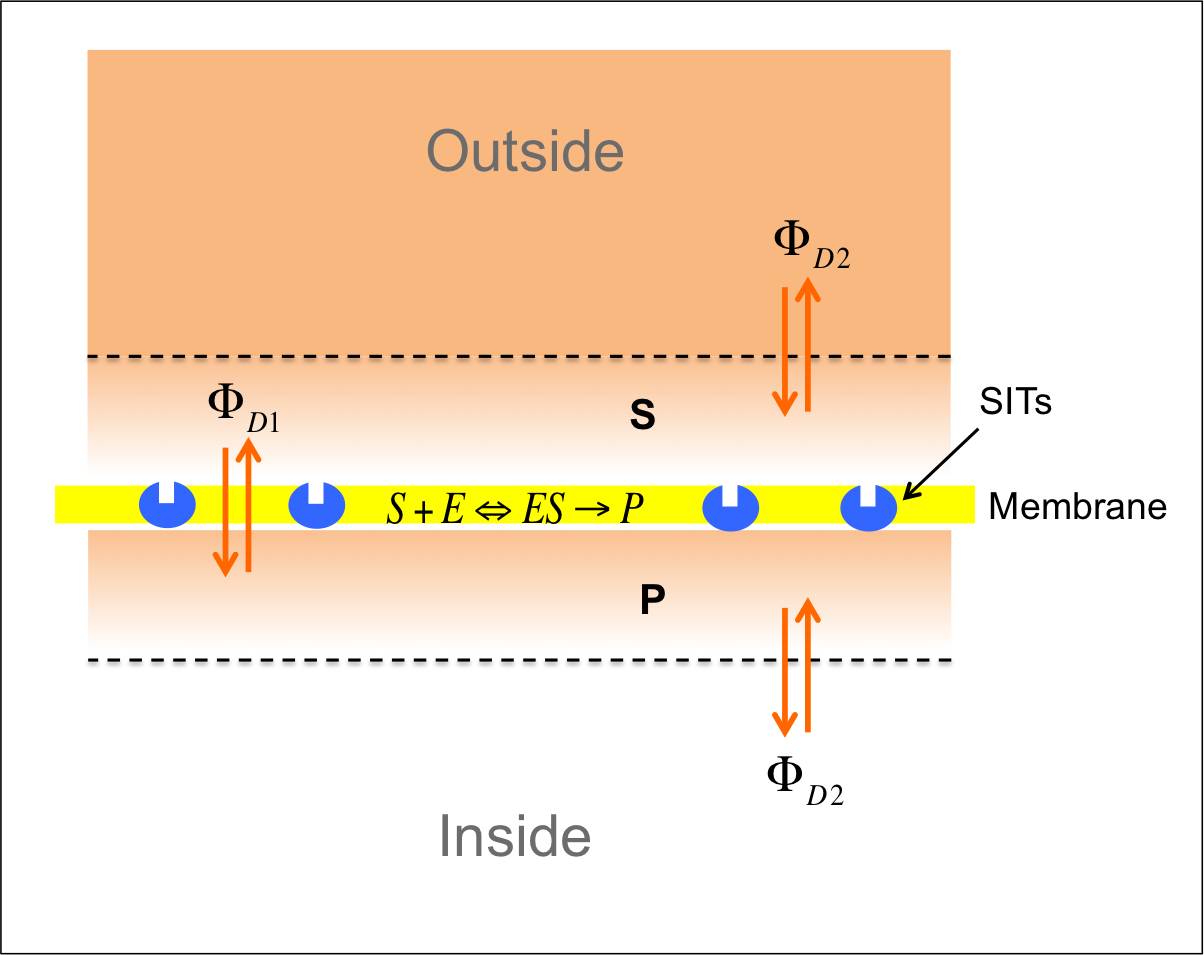


**Figure S1. Two types of diffusion flux in nutrients dynamics.**

$\Phi_{D1}$is the flux of diffusion through membrane. $\Phi_{D2}$is the diffusion flux that controls concentrations of substrate and product near the membrane, where the enzymatic reaction occurs.

Figure S1 is a schematic graph of two types of diffusion fluxes in nutrients dynamics. The first flux ($\Phi_{D1}$) is the transportation through membrane. Since diatoms uptake silicic acid mostly in the neutral molecular form [40],[41] it is possible for the small molecules of silicic acid to transport through the membrane. The second flux ($\Phi_{D2}$) supplies/depletes the materials (substrate or product) to/from the enzymatic reaction sites. The effect of these fluxes on the total nutrient dynamics is discussed below.

- $\Phi_{D1}$: Transport through a membrane:

Monosilicic acid is solvable in water and the neutral form of it can diffuse across cell membrane. Therefore, we have to determine the diffusion rate and consider its contribution to the transport process. Lipid membrane permeability to mono silicic acid for inner mitochondrial membrane was calculated by [53] using the data by [54] to be $10^{-9}m s^{-1}$. They also calculated permeability of cell membrane to mono silicic acid using the model of [55] to be $10^{-10}m s^{-1}$ [56]. The diffusion rate through a membrane is given by a modification of Fick’s law, which states that the diffusion rate is proportional to the permeability coefficient P, to the surface area of the membrane and to the difference in solution concentration in two sides of the membrane [S1]:

$\frac{dn}{dt}=PA\Delta C$. (S1-1)

Using the value of estimated P and the surface area of diatom cell [table 1], we calculate the diffusion rate for different concentrations of silicic acid. For $\Delta C=10 \mu mol L^{-1}$ the diffusion rate is approximately $10^{-4} fmol cell^{-1}h^{-1}$ and for $\Delta C=500 \mu mol L^{-1}$it is approximately $5\times10^{-3} fmol cell^{-1}h^{-1}$. The observed uptake rate of the diatom after a short time (2min) in data from [50] for the same concentrations of silicic acid is around $5 fmol cell^{-1}h^{-1}$ and $400 fmol cell^{-1}h^{-1}$ respectively. The difference between the rate by which diffusion across membrane can provide the substrate and the observed uptake rate is up to five orders of magnitude. Therefore, although the cell membrane is semi-permeable to silicic acid, still this process is too slow for the high demand of silicon and low concentrations of silicic acid in the medium.

- $\Phi_{D2}$: Supplying/depleting materials:

To investigate the effect of diffusion as a supplying/depleting mechanism diffusion has been included in the MM kinetics equation. Bonachela et. al. [45] have derived the following equation for the uptake rate versus substrate concentration,

where D is diffusion coefficient of substrate, $K_{s}$ and $V_{\max}$, Michaelis-Menten coefficients and $r_{c}$ is the cell radius (assuming a spherical cell). $\tilde{K}_{s}$is the modified half-saturation coefficient. This equation describes diffusion-mediated MM kinetics.


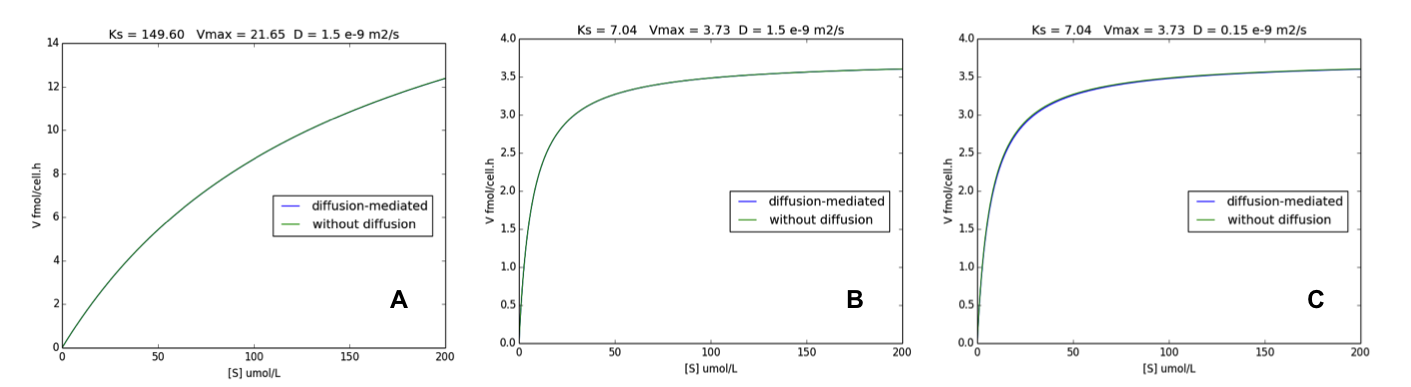


**Figure S2. Comparison of the rates of diffusion-mediated uptake with no-diffusion uptake**

(A) and (B) uptake rates for two observed values of MM coefficients. The curves are almost the same. (C) The same as B with diffusion constant one order of magnitude smaller. The effect of diffusion is still negligible.

Using the values for silicic acid and diatom cell (table 1) and the $K_{s}$and $V_{\max}$values measured in different incubation times [50], we plot the diffusion-mediated MM kinetics and compare it with MM kinetics without diffusion. We have used the $K_{s}$and $V_{\max}$ at t=30 min and t=3 h. But for any value in the range of measured values for T. pseudonana the conclusion is the same. As it is plotted in Figure S2, the diffusion term does not have a significant effect on the kinetics. Even with the value of diffusion coefficient of silicic acid one order of magnitude smaller, still the effect is negligible.

- Discussion:

Based on the data and calculations above, none of the two diffusion types can control the dynamics of transport significantly. We therefore, ignore the diffusion in our model and apply the MM-type kinetics as the main transport method for modeling synchronized diatom cells.

Since passive diffusion cannot be accounted for the high rates of uptake observed with those cells that did not experience a long pre-starvation, the linear uptake dynamics in that case still remains an interesting discussion. One candidate for explaining linear data could be silicic acid transport across the membrane when possibly being facilitated by a hydrophobic compound that binds to silicic acid. In that case, membrane diffusion rates could be substantially increased.  The basis for this idea comes from a study characterizing an apparent silicon ionophore from a diatom that facilitated transport across an organic layer [S2].

Another relevant consideration is that the averaging over a population of cells that are in different cell cycle phases is potentially a risky oversimplification. When dealing with non-synchronized cells, the uptake rate of each cell is not simply the average. We have formulated this point mathematically through equations (1) to (5). In the case of most cells being synchronized, after applying an approximation, eq. (5) is derived. Eq. (5) can be rewritten as:

$\frac{dC_{u}}{dt}=-\frac{1}{N}\frac{dC_{m}}{dt}-C_{u}\frac{dN}{dt}$ . (S1-3)

Knowing and (amount of consumed silicon and number of cells), this equation is a first order differential equation to be solved for calculating uptakes. It considers an approximation for the effect of non-synchronized cells. Since $\frac{dC_{m}}{dt}$is negative and $\frac{dN}{dt}$ is positive in most cultures of interest the uptake rate, which is the left-hand-side of eq. (S1-3), is smaller than the average (the first term). The effect of the second term in the case of our data is shown in Figure 9. Consequently, it could also be possible that this term is responsible at least for some part of the very large value and non-saturable uptake rates observed in non-synchronized (short pre-starvation) populations. Whether this modification is slight or significant should be investigated with the original data.

References

S1. Van't Riet K, Tramper J (1991) Basic bioreactor design. CRC Press.

S2. Bhattacharyya P, Volcani BE (1983) Isolation of silicate ionophore(s) from the apochlorotic diatom Nitzschia, alba. Biochemical and biophysical research communications 114(1): 365-372.
