# Supplementary material for: Understanding the Sub-Cellular Dynamics of Silicon Transportation and Synthesis in Diatoms Using Population-Level Data and Computational Optimization
Source: PLoS Comput Biol. 2014 Jun 19;10(6):e1003687. doi: 10.1371/journal.pcbi.1003687 (PMC4063665; doi:10.1371/journal.pcbi.1003687)
Supplement: Text S2 — Intracellular dynamics system of equations. (DOCX) [file pcbi.1003687.s004.docx]

**Supplementary Materials: Text S2**

Intracellular dynamics system of equations

Using the following definitions for variables, $\mathbf{Y}$, initial values, $\mathbf{Y}_{0}$ and parameters, $\mathbf{P}$,

$\mathbf{P}=\left(\begin{matrix} \frac{k_{2}}{k_{1}} \\ k_{2}\cdot E_{1f}(t_{0}) \\ E_{1f}(t_{0}) \\ \frac{k_{4}}{k_{3}} \\ k_{4}\cdot E_{2f}(t_{0}) \\ E_{2f}(t_{0}) \\ \frac{k_{6}}{k_{5}} \\ k_{6}\cdot E_{3f}(t_{0}) \\ E_{3f}(t_{0}) \end{matrix}\right)$; $\mathbf{Y}=\left(\begin{matrix} C_{u} \\ E_{1f} \\ E_{1}C_{m} \\ C_{p} \\ E_{2f} \\ E_{2}C_{p} \\ C_{s} \\ E_{3f} \\ E_{3}C_{s} \\ C_{d} \\ C_{m} \end{matrix}\right)$; $\mathbf{Y}_{\mathbf{0}}=\left(\begin{matrix} 0 \\ p_{3} \\ 0 \\ 0 \\ p_{6} \\ 0 \\ 0 \\ p_{9} \\ 0 \\ 0 \\ C_{m0} \end{matrix}\right)$; (S2-1)

equations (S2-2)-(S2-12) describes the time derivatives of concentrations.

(S2-2)

(S2-3)

(S2-4)

(S2-5)

(S2-6)

(S2-7)

(S2-8)

(S2-9)

(S2-10)

(S2-11)

(S2-12)

Appearance of cell population, N (cells/L), makes dimensional balance for two sides of eq. (S2-12). In some works [e.g. 45] the inverse of cell volume (L/cell) appears in the first term of this equation, which, even though balances dimensions of two sides, it does not correspond to the real case; because silicon in environment reacts with enzymes (SITs here) which are located on the membrane and the cell volume is not reachable for $C_{m}$.

In order to solve eqs. (S2-2)-(S2-12) numerically, we first made them dimensionless. This is important in order to provide a smaller search space for optimizing and therefore, a higher precision in searching within a specific amount of time. We have used the dimensionless transformation for basic quantities, mol value, volume and time, as follow,

$m'=\frac{m}{10^{-15}mol}; v'=\frac{v}{10^{-14}L}; t'=\frac{t}{1 \min};$ (S2-13)

and every other quantity can be derived from above equation. For representing outputs of the model, we perform the inverse calculation.
